# Prof. Van Buren on the Genito-Urinary Organs and Syphilis

**Published:** 1874-07

**Authors:** 


### Bibliographical.

A Practical Treatise on the Surgical Diseases of the Genito-Urinary Organs, Including Syphilis. Designed as a manual for students and practitioners. With engravings and cases. By W. H. Van Buren, A.M., M.D., Professor of the Principles of Surgery, with Diseases of the Genito-Urinary System and Clinical Surgery, in Bellevue Hospital Medical College, etc., and E. L. Keys, A.M., M.D., Professor of Dermatology, in Bellevue Hospital Medical College; Surgeon to the Charity Hospital, Venereal Division, etc., New York: D. Appleton & Co., 1874. Octavo, cloth, pp. 672. Price $5.00.

Of the making of books there is no end, is the adage, trite as it is true; of the making of *good* books, such as we esteem to be the one now in hand, there ought to be no end. The many complete monographs with which the medical press is now yearly blessing our profession we regard as gratifying evidence of the rapidly advancing knowledge of the science and arts of medicine.

We have, at present, neither time nor space for so full a review of the contents of this excellent monograph as we would gladly give to our readers. The commanding reputation of Dr. Van Buren in this specialty, and of the great school and hospital from which he has drawn his clinical materials, together with the general interest which attaches to the subject-matter itself, will, we trust, lead very many of those for whom it is our office to cater to possess themselves at once of the volume and form their own opinions of its merit.

At page 24 and onward, we find a very satisfactory account of chronic circumscribed inflammation of the penis, manifesting itself in a hardened button within the corpus cavernosum, and causing the organ to curve strongly to the affected side in erection, which we do not remember to have seen elsewhere than in the medical periodicals.

The directions for catheterism are concisely and clearly given and well illustrated by cuts. Very many otherwise excellent practitioners would profit by a careful reading under this head. The subjects of gonorrhœa, gleet, and the complications of gonorrhœa are well handled, and a selection of formulæ given. The subject of stricture is very fully and, we think, admirably treated. The same remark is equally applicable to prostatic hypertrophy. As a soothing injection in the latter disease the chlorate of potassium is mentioned, the biborate of sodium particularly recommended, and carbolic acid condemned. In our own very limited observation a saturated solution of the chlorate has proven far the best, and we heartily unite in the opinion that carbolic acid, in any strength, "does not yield good results."

Stone in the bladder receives a full share of attention, and lithotomy and lithotrity brought up to the latest improvements with ample illustrations. Next follow the diseases of the kidney and of the scrotum and testicle, followed by impotence, self-abuse, pollution, spermatorrhœa and diseases of the cord.

Part second, comprising about one-fourth of the volume, treats of syphilis and chancroid in a clear and satisfactory manner, with the latest improvements. For the destruction of chancroids the author mentions three caustics, the actual and two potential, of which nitric acid is esteemed the best, whilst the nitrate of silver is not even alluded to. The thorough destruction of the ulcers is insisted on, and *strong* nitric acid is necessary to best effect this. All of which is as it should be. When it is not advisable to use the caustic application, ointments are pointedly condemned, and a dressing of powdered iodoform recommended.

In true syphilis our author remarks: "In the early manifestations of syphilis mercury is specially potent. Under its kindly influence the chancre heals, the early eruptions fade." "The iodides have but little power over early syphilis," etc. "Salivation is harmful." "In all cases where the diagnosis is certain, of a late, tertiary, syphilitic symptom requiring iodide of potassium, continue increasing the dose until the symptom yields or the patient will bear no more."

